# Octahedron Iron Oxide Nanocrystals Prohibited *Clostridium difficile* Spore Germination and Attenuated Local and Systemic Inflammation

**DOI:** 10.1038/s41598-017-08387-y

**Published:** 2017-08-15

**Authors:** Wei-Ting Lee, Ya-Na Wu, Yi-Hsuan Chen, Shang-Rung Wu, Tsai-Miao Shih, Tsung-Ju Li, Li-Xing Yang, Chen-Sheng Yeh, Pei-Jane Tsai, Dar-Bin Shieh

**Affiliations:** 1 Institute of Basic Medical Sciences, National Cheng Kung University, 1 University Road, Tainan, 701 Taiwan; 2 Institute of Oral Medicine, National Cheng Kung University, 1 University Road, Tainan, 701 Taiwan; 3 Department of Medical Laboratory Science and Biotechnology, National Cheng Kung University, 1 University Road, Tainan, 701 Taiwan; 4 Department of Chemistry, National Cheng Kung University, 1 University Road, Tainan, 701 Taiwan; 5 Center of Infectious Disease and Signaling Research, National Cheng Kung University, 1 University Road, Tainan, 701 Taiwan; 6 Department of Stomatology, National Cheng Kung University Hospital, College of Medicine, National Cheng Kung University, 138 Sheng-Li Road, Tainan, 704 Taiwan; 7 Advanced Optoelectronic Technology Center and Center for Micro/Nano Science and Technology, National Cheng Kung University, 1 University Road, Tainan, 701 Taiwan

## Abstract

Clinical management of *Clostridium difficile* infection is still far from satisfactory as bacterial spores are resistant to many chemical agents and physical treatments. Certain types of nanoparticles have been demonstrated to exhibit anti-microbial efficacy even in multi-drug resistance bacteria. However, most of these studies failed to show biocompatibility to the mammalian host cells and no study has revealed *in vivo* efficacy in *C*. *difficile* infection animal models. The spores treated with 500 µg/mL Fe_3-δ_O_4_ nanoparticles for 20 minutes, 64% of the spores were inhibited from transforming into vegetative cells, which was close to the results of the sodium hypochlorite-treated positive control. By cryo-electron micro-tomography, we demonstrated that Fe_3-δ_O_4_ nanoparticles bind on spore surfaces and reduce the dipicolinic acid (DPA) released by the spores. In a *C*. *difficile* infection animal model, the inflammatory level triple decreased in mice with colonic *C*. *difficile* spores treated with Fe_3-δ_O_4_ nanoparticles. Histopathological analysis showed a decreased intense neutrophil accumulation in the colon tissue of the Fe_3-δ_O_4_ nanoparticle-treated mice. Fe_3-δ_O_4_ nanoparticles, which had no influence on gut microbiota and apparent side effects *in vivo*, were efficacious inhibitors of *C*. *difficile* spore germination by attacking its surface and might become clinically feasible for prophylaxis and therapy.

## Introduction

Nanomaterials have attracted significant interest in medicine. Certain microorganism-reactive nanomaterials have been used as alternative bactericides^1^, namely, silver, zinc oxide, and titanium oxide nanoparticles, all of which have remarkable antibacterial properties^2, 3^. The antibacterial mechanisms of nanoparticles may be attributable to their generation of reactive oxygen species, disruption of cell membranes, ability to bind thiol groups, and their release of toxic ions^4^. Spore-formation enables bacteria to survive nutritional deprivation and harsh environments. They can resist ultraviolet radiation, desiccation, high temperatures, extreme freezing, and chemical disinfectants^5^. Spores can reactivate themselves to the vegetative state when the environment becomes favorable. Therefore, *Clostridium* species, spore-forming pathogens, usually challenge clinical disease management and prevention. *Clostridium difficile*, a pathogen associated with healthcare-facility-related (nosocomial) infections, is a major cause of antibiotic-treatment-related diarrhea, pseudomembranous colitis, abdominal pain, fever, and death^6^. The normal flora in the gut can inhibit the growth of *C*. *difficile* and therefore protect patients from developing *C*. *difficile* infection^7^. *C*. *difficile* infection usually occurs in patients on a long-term regimen of antibiotics, and it is often initiated by the spores acquired from healthcare workers^8, 9^. Once a patient develops *C*. *difficile* infection, there are only a few antibiotics available to control it^10^. Moreover, the failure rate of first-line antibiotics and the *C*. *difficile* infection relapse rate are both dramatically high^10, 11^. Consequently, about two decades ago, the attributable post-diagnosis mortality rate was 6.9% at 30 days and 16.7% at 1 year^12^. The spores of *C*. *difficile* are the major cause of *C*. *difficile* infection. Compared with oxygen-sensitive vegetative bacteria, *C*. *difficile* spores survive for up to several months in room air and in low-pH gastric contents^13^. As the spores enter the human digestive tract, they germinate after they have been exposed to bile salts and their derivatives, and then they are colonized in the colon^14^. The virulence of *C*. *difficile* depends upon the gene expression of *tcd*A-encoded toxin A, an enterotoxin, and *tcd*B-encoded toxin B, a cytotoxin^15^. Both cause intestinal inflammation and neutrophil infiltration in the infected foci^16, 17^.

The incidence of *C*. *difficile* infection has significantly increased in the past 15 years^18^. *C*. *difficile* infection has become a major cause of nosocomial-associated infection in the world^9^. Antibiotic-resistant *C*. *difficile* is not only potentially fatal, but it also causes healthcare-associated economic burdens^19^. The available present antibiotics are targeted to vegetative bacterium, however, the infective form is the spore. Current *C*. *difficile* infection clinical management is still far from satisfactory because the spores are resistant to many chemical agents and physical treatments, which makes effective management of the spores an important problem^20^. Therefore, anti-germination approach could lead to the prevention of *C*. *difficile* infection. Some newly designed cholate derivatives show promise against *C*. *difficile* infection; however, they are still under pre-clinical study^21, 22^. Sodium hypochlorite, a standard disinfectant, has outstanding antimicrobial activity but undesirable side effects: it is corrosive and irritates tissue^5^. To control spore germination and *C*. *difficile* infection, it is important to develop an efficacious and biocompatible spore-control strategy.

There are various well-known antibacterial nanomaterials, e.g., silver (Ag) and zinc oxide (ZnO) nanoparticles^23, 24^, and zero-valent iron nanoparticles, which are prominently bactericidal against *Escherichia coli* ^25^. The nanotechnology even has been reported that it could overcome the problem of multi-drug resistant bacteria^26^. However, most current antibacterial nanomaterials are primarily multifunctional generic biocidal agents against vegetative cells; their sporicidal activity at high concentrations has been explored in only a few studies^27, 28^. Many studies demonstrated the excellent antibacterial ability of nanoparticles, but their impacts to the gut microbiota were not clear. The healthy gut microbiota prevent the host from colonic pathogen^29^. Therefore, an ideal antibacterial nanomaterial should exhibit the specific pathogen targeting capability without causing disruption of healthy gut microibota.

The toxicity of various bactericidal nanoparticles to mammalian cells has become an increasing concern. Iron-containing particles are generally recognized as highly biocompatible nanomaterials. A few specific iron oxide nanoparticles have recently been reported to be antibacterial; they were synthesized and showed therapeutic potential for cancer therapy^30, 31^. In this study, we evaluated whether such single-crystal nonstoichiometric Fe_3-δ_O_4_ magnetite nanoparticles could inhibit *C*. *difficile* spore germination *in vivo*. The potential mechanisms were explored from both material science and molecular biology perspectives.

## Results

### Testing the sporicidal activity of Fe_3-δ_O_4_ nanoparticles against *C. difficile*

We used a previously described method^32^ to explore the inhibitory efficacy of Fe_3-δ_O_4_ nanoparticles to spores. After they had been incubated with sodium hypochlorite for 20 minutes, inactivated spores were used as positive controls, as described elsewhere^33^. After they had been incubated with nanomaterials of various concentrations (5, 50, and 500 μg/mL) for 20 minutes, the treated spores were then stimulated using 10 mM taurocholate to induce germination.

**Figure 1:**
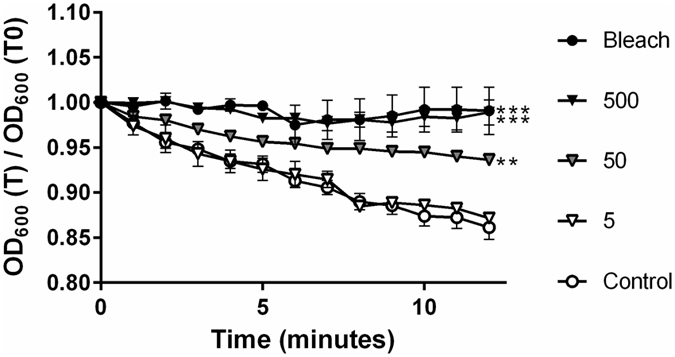
*C*. *difficile* spore germination was significantly inhibited in spores treated with Fe_3-δ_O_4_ nanoparticles. Purified CCUG 37780 spores were incubated in BHIS medium containing Fe_3-δ_O_4_ nanoparticles ([500 µg/mL (▲), 50 µg/mL (), or 5 μg/mL (▵)]), or 3% sodium hypochlorite as a positive control. The kinetics of spore germination was analyzed using spectrometric absorption referenced to the starting point. OD_600_(T) = different time points after taurocholate treatment; OD_600_(T_0_) = time zero. Spore germination was significantly inhibited in spores treated with Fe_3-δ_O_4_ nanoparticles. (***P < 0.001; one-way analysis of variance [ANOVA] followed by Tukey’s Multiple Comparison test).

**Figure 2:**
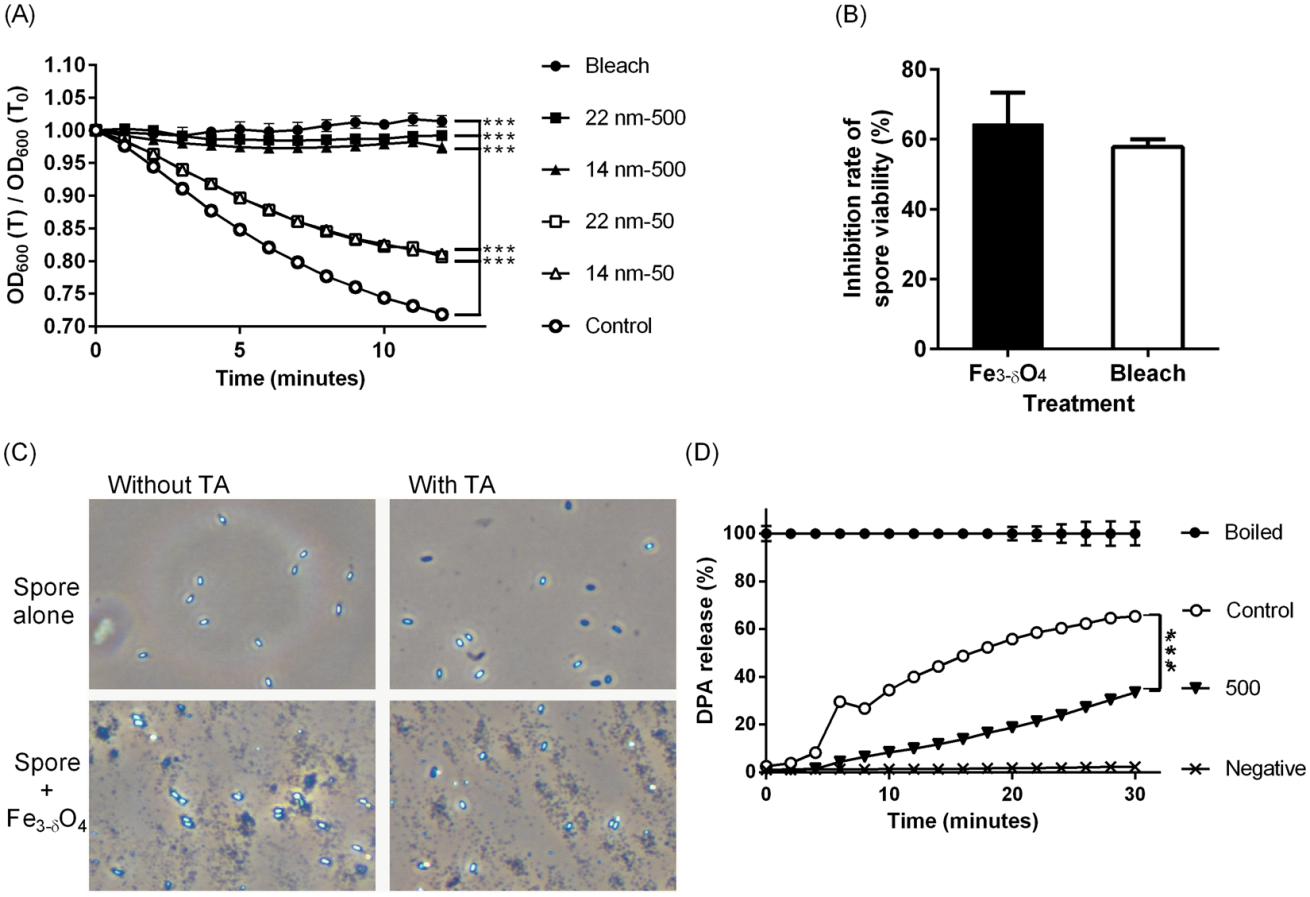
The viability and germination inhibition of *C*. *difficile* CCUG 37780 spores by Fe_3-δ_O_4_ nanoparticles were dose-dependent rather than size-dependent. (**A**) The spores were first treated for 20 minutes with 500 μg/mL of 22-nm Fe_3-δ_O_4_ (■), 500 μg/mL of 14-nm Fe_3-δ_O_4_ (▲), 50 μg/mL of 22-nm Fe_3-δ_O_4_ (□), 50 μg/mL of 14-nm Fe_3-δ_O_4_ (▵), or 3% sodium hypochlorite (●) and then were treated with taurocholate to induce germination. Both 14-nm and 22-nm Fe_3-δ_O_4_ nanoparticles had a similar dose-dependent effect on spore germination. (**B**) After *C*. *difficile* spores and 500 μg/mL of 22-nm Fe_3-δ_O_4_ nanoparticles or 3% sodium hypochlorite had been incubated for 20 minutes, the spores were plated on BHIS agar for a colony formation assay the next day. The level of colony-forming unit inhibition was similar for Fe_3-δ_O_4_ nanoparticle- and sodium hypochlorite-treated spores. (**C**) The spores were treated for 20 minutes with 500 μ﻿g/mL of 22-nm Fe_3-δ_O_4_ and then stimulated using 10 mM taurocholate. After 15 minutes, the phase contrast of the spores was recorded under a phase contrast microscope. (**D**) The DPA-release assay showed that Fe_3-δ_O_4_ nanoparticles-treated spores released less DPA than did the control group. Data are mean ± SEM. (***P < 0.001; one-way analysis of variance (ANOVA) followed by Tukey’s Multiple Comparison test).

Spores treated with 50 and 500 μg/mL of Fe_3-δ_O_4_ nanoparticles showed significantly (**P < 0.01 and ***P < 0.001) inhibited germination (Fig. 1), but spores treated with 5 μg/mL did not. There were no significant differences between the sodium hypochlorite-treated positive controls and the Fe_3-δ_O_4_ nanoparticle-treated (500 μg/mL) spores (Fig. 1). Intriguingly, there was no significant difference in germination inhibition between spores treated with 14-nm and 22-nm Fe_3-δ_O_4_ nanoparticles (Fig. 2A). In Supplementary Table 1, the germination kinetic results show that the Michaelis-Menten constant (*Km*) for taurocholate-treated *C*. *difficile* CCUG 37780 spores increased from 4.34 to 8.43 M in mice treated with 50 ﻿μg/mL of Fe_3-δ_O_4_ nanoparticles. The kinetic analysis suggested that Fe_3-δ_O_4_ nanoparticles have an inhibiting constant (*Ki*) of approximately 62 μg/mL. To confirm the sporicidal property of 500 μg/mL of Fe_3-δ_O_4_, the number of Fe_3-δ_O_4_- and 3%-sodium hypochlorite-treated colony-forming unit (CFU) spores was counted. Compared with the negative control set, the number of CFUs of Fe_3-δ_O_4_-treated and 3%-sodium hypochlorite-treated spores was ~60% lower (Fig. 2B). Furthermore, the inhibition rates of CFUs in Fe_3-δ_O_4_-treated and in 3%-sodium hypochlorite-treated spores were not significantly different.

Phase-contrast microscopy showed that in the positive controls, to which taurocholate was added for 15 minutes, but not in the negative controls, the contrast of the spores transitioned from bright to dark (Fig. 2C). The contrasts of the spores treated with 500 μg/mL Fe_3-δ_O_4_ nanoparticles were not significantly different. The luminescence signal that accompanies DPA release was significantly higher during taurocholate-induced spore germination (Fig. 2D). However, the luminescence signal was less intense and less abundant in the Fe_3-δ_O_4_-treated group than in the taurocholate-treated group. No luminescence signal was detected in the group of spores not treated with taurocholate because the spores did not release DPA.

### Inhibitory effect of Fe_3-δ_O_4_ nanoparticles on virulent strains of *C. difficile*

**Figure 3:**
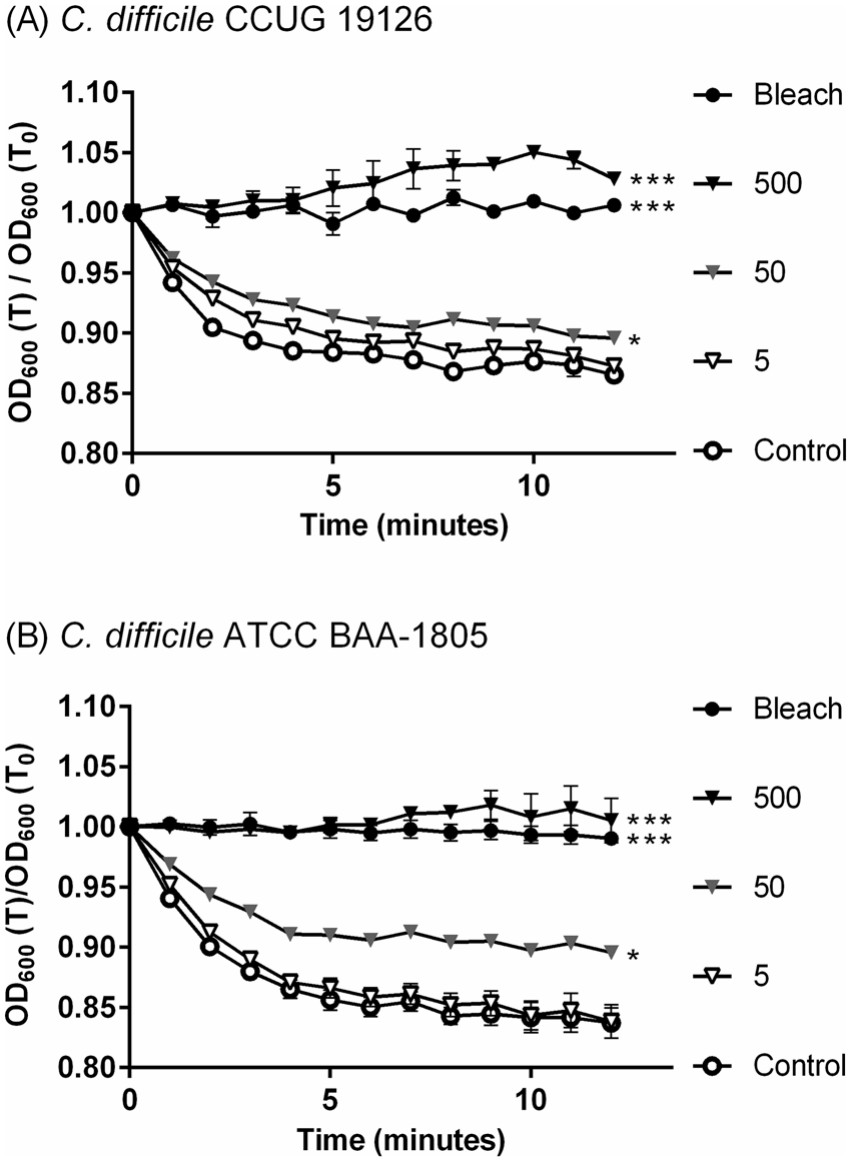
Spore germination was significantly inhibited in Fe_3-δ_O_4_-treated pathogenic strains of *C*. *difficile*. Spores from two pathogenic strains of *C*. *difficile* (CCUG 19126 and ATCC BAA-1805) were treated for 20 minutes with various concentrations of Fe_3-δ_O_4_ nanoparticles and then with 10 mM taurocholate to induce spore germination. Spore germination was effectively inhibited in the two Fe_3-δ_O_4_-treated pathogenic strains, as it was in the nonpathogenic CCUG 37780 strain. (***P < 0.001, *P < 0.05; one-way analysis of variance [ANOVA] followed by Tukey’s Multiple Comparison test).

*C*. *difficile* strain CCUG 37780 does not have the *tcdA* and *tcdB* genes^34^. To determine what doses of Fe_3-δ_O_4_ nanoparticles affect the spore germination of *C*. *difficile*, CCUG 19126 and BAA-1805 *tcdA*- and *tcdB*-positive strains were treated for 20 minutes with 5, 50, and 500 μg/mL doses of Fe_3-δ_O_4_ nanoparticles, and then treated with 10 mM taurocholate. The germination of *C*. *difficile* CCUG 19126 spores and of *C*. *difficile* BAA-1805 spores was not significantly different between those treated with 500 μg/ml of Fe_3-δ_O_4_ nanoparticles and those treated with sodium hypochlorite, but CCUG 19126 was relevantly resistant to treatment with 50 μg/mL of Fe_3-δ_O_4_ nanoparticles (Fig. 3A).

**Figure 4:**
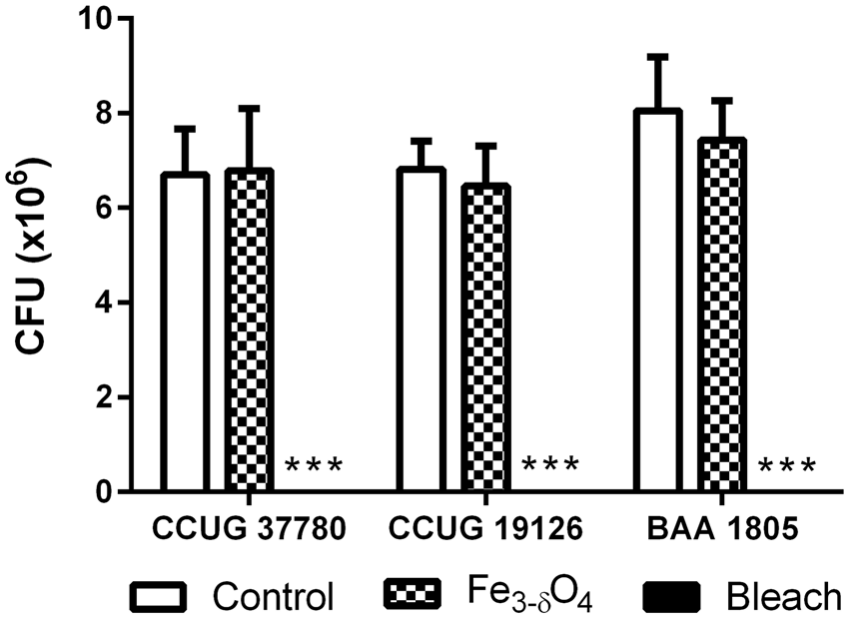
The viability of *C*. *difficile* vegetative cells was not significantly affected by Fe_3-δ_O_4_-treatment. Fe_3-δ_O_4_ nanoparticles (500 µg/mL) did not inhibit the viability of vegetative *C*. *difficile* in all three test strains compared cells treated with 3% sodium hypochlorite. (***P < 0.001; Student’s *t* test).

To evaluate the bactericidal effect of Fe_3-δ_O_4_ nanoparticles on vegetative cells in different strains, three groups of vegetative cells—CCUG 37780, CCUG 19126, and BAA-1805—were first treated for 20 minutes with 500 μg/mL of Fe_3-δ_O_4_ nanoparticles, spread on BHIS agar plates, and their CFUs were counted. The growth behavior of the Fe_3-δ_O_4_-treated vegetative cells was not significantly different from that of the negative control groups; however, almost all the vegetative cells treated with 3% sodium hypochlorite died (Fig. 4). Treatment with Fe_3-δ_O_4_ nanoparticles did not damage *C*. *difficile* vegetative cells.

### The mechanisms underlying Fe_3-δ_O_4_-inhibited spore germination

To determine whether any additional underlying mechanisms allow Fe_3-δ_O_4_ nanoparticles to inhibit spore germination, we first excluded the sporicidal activity of the coating polymer poly-(styrene-alt-maleic acid) (PSMA) on Fe_3-δ_O_4_ nanoparticles. At a concentration higher than that on PSMA-coated on Fe_3-δ_O_4_ nanoparticles, PSMA did not inhibit spore germination (Supplementary Fig. 1). In addition, to verify whether Fe_3-δ_O_4_ nanoparticles promoted or hindered the germination-inducing activity of taurocholate, 10 mM taurocholate was incubated for 20 minutes with different concentrations of Fe_3-δ_O_4_ nanoparticles and then used to treat spores. The taurocholate incubated with Fe_3-δ_O_4_ nanoparticles still induced *C*. *difficile* spore germination (Supplementary Fig. 2).

**Figure 5:**
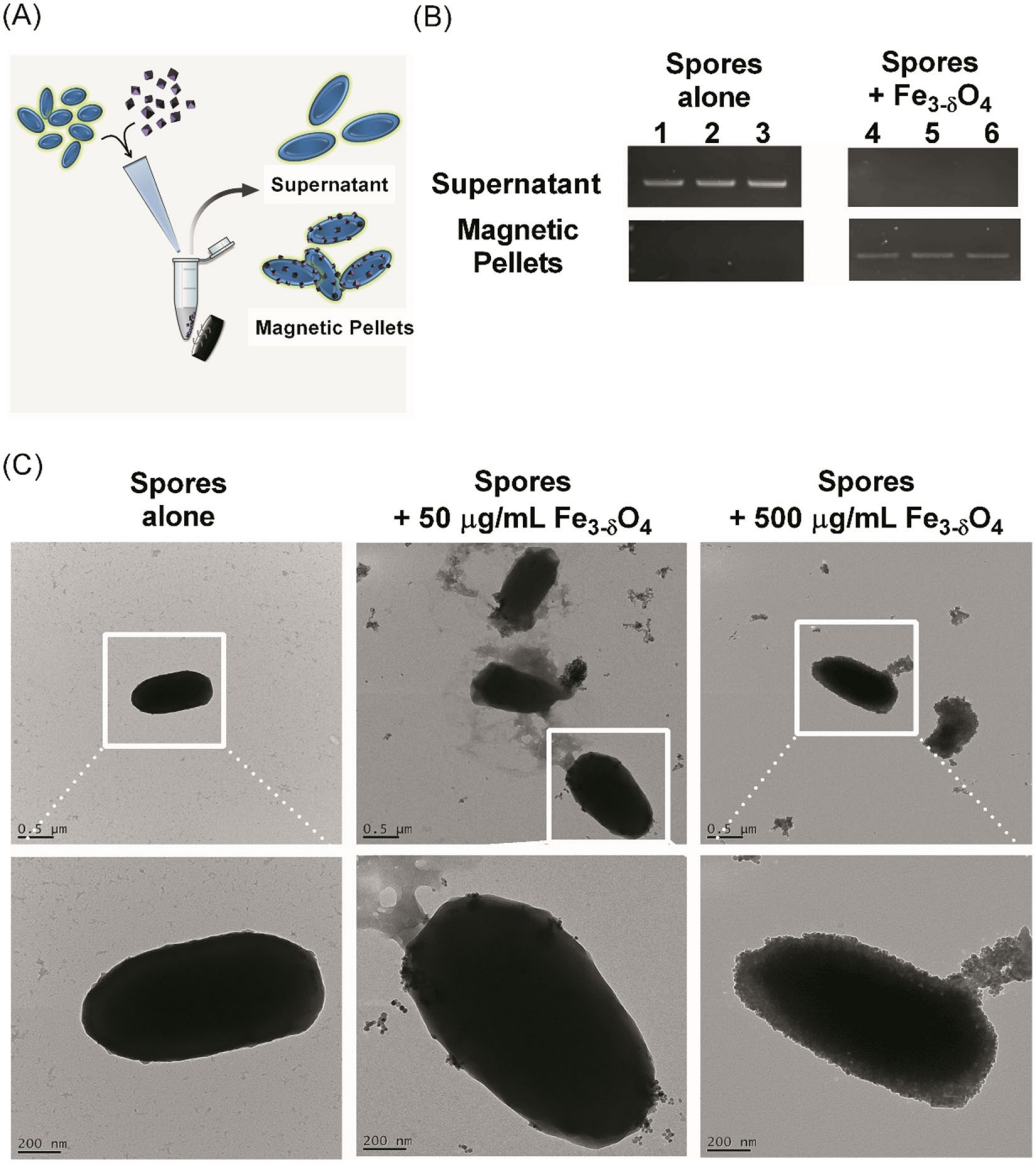
Fe_3-δ_O_4_ nanoparticles bound to the surface of *C*. *difficile* (ATCC BAA-1805) spores, which allowed magnetic attraction. (**A**) Schematic illustration of the magnetic separation of spores with which Fe_3-δ_O_4_ nanoparticles had bound: these spores were attracted by a magnet, but spores with which the nanoparticles had not bound were retained in the supernatant. (**B**) The spores were incubated for 20 minutes with Fe_3-δ_O_4_ nanoparticles, and then *tcdB* genes were isolated from the supernatant or the magnetically concentrated precipitates were detected after PCR amplification. Lanes 1–3 are spores alone and lanes 4–6 are spores treated with 500 µg/mL of Fe_3-δ_O_4_ nanoparticles. TcdB amplicons were detected in the supernatant of the control group, but they were found only in the magnetically concentrated fraction after they had been treated with Fe_3-δ_O_4_ nanoparticles. (**C**) TEM images of the spores (upper panel: 10,000×; lower panel: 30,000×). The left image shows that native spores have a smooth coat; accumulated Fe_3-δ_O_4_ nanoparticles on spore surfaces are evident after incubation. The spores treated with 500 µg/mL were completely covered by the Fe_3-δ_O_4_ nanoparticles.

To identify any additional underlying mechanisms that allow Fe_3-δ_O_4_ nanoparticles to inhibit spore germination, we took advantage of the magnetic property of Fe_3-δ_O_4_. Some *C*. *difficile* BAA-1805 spores were incubated with 500 μg/mL of Fe_3-δ_O_4_ nanoparticles for 20 minutes and then separated, using a magnet, into two parts: the nonmagnetic supernatant and the magnetic pellets (Fig. 5A). Each part was collected, and PCR was used to detect the *tcdB* gene. The *tcdB* gene was detected only in the supernatant (Fig. 5B) in the spores-alone groups. In the Fe_3-δ_O_4_-treated spores, the *tcdB* gene was also detected in the magnetic pellets.

To clarify the interaction between Fe_3-δ_O_4_ and spores, the Fe_3-δ_O_4_-treated spores were examined under a TEM. The TEM images showed that spores treated with 50 μg/mL of Fe_3-δ_O_4_ nanoparticles were only spotted with Fe_3-δ_O_4_ nanoparticles, but that spores treated with 500 μg/mL were almost fully surrounded (Fig. 5C). To reconfirm that Fe_3-δ_O_4_ nanoparticles were simply attached to the surface of the spores instead of diffused into the spores, the spores were examined using cryo-electron tomography. The rotated dynamic video (Supplementary Video 1) revealed that Fe_3-δ_O_4_ nanoparticles were directly and dose-dependently bound to the surface of *C*. *difficile* spores.

### Fe_3-δ_O_4_ nanoparticles as an *in vivo* sporicidal agent against *C. difficile* infection

**Figure 6:**
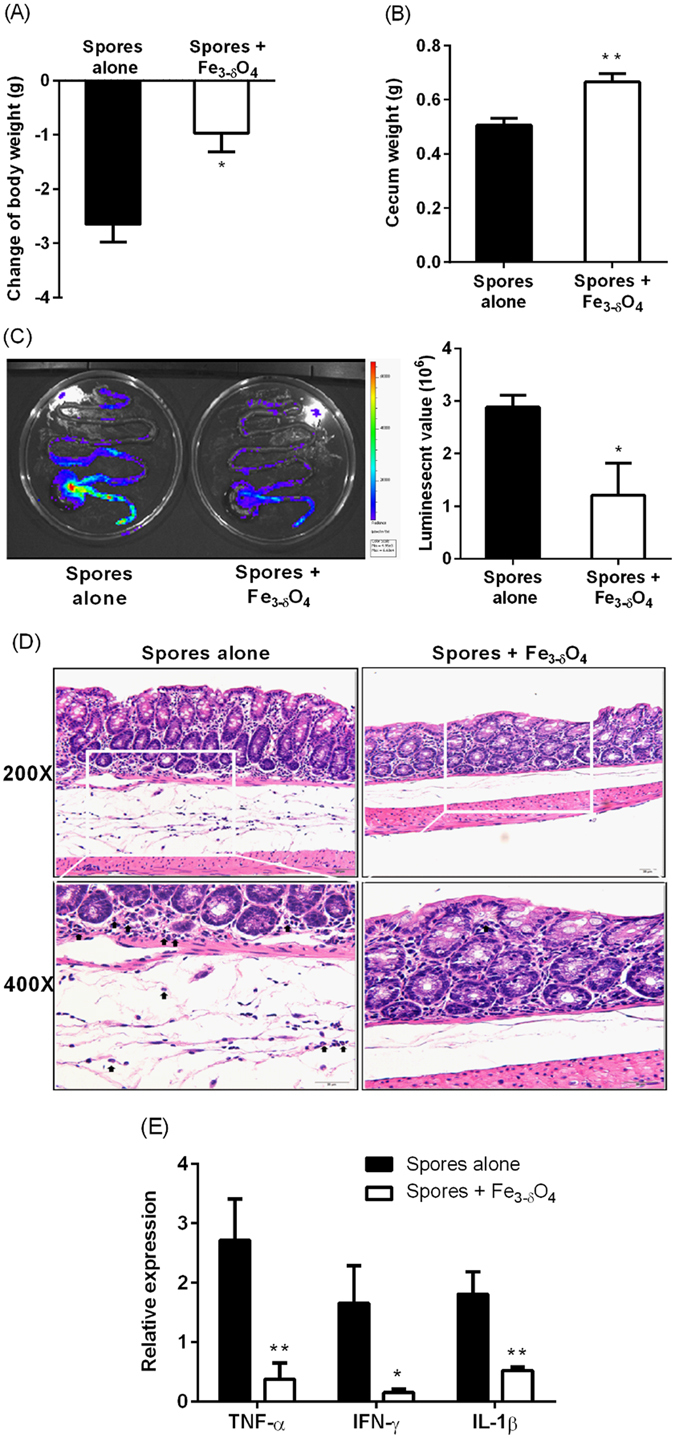
*C*. *difficile* infection-induced colitis was attenuated in mice treated with Fe_3-δ_O_4_ nanoparticles. Purified CCUG 19126 spores were incubated for 20 minutes with Fe_3-δ_O_4_ nanoparticles, and then the mice were injected with them. Three days later, the mice were killed. (**A**) The mean bodyweight-loss of the Fe_3-δ_O_4_ nanoparticle-treated mice was significantly lower than that of the spores-alone control mice. (**B**) The mean weight-loss of the cecums of the Fe_3-δ_O_4_ nanoparticle-treated mice was also significantly lower than that of spores-alone control mice. (**C**) The inflammation of the isolated colon from 11FNL/FVB/NJ transgenic mice with NF-κB-signal-activated bioluminescence was detected. The left image shows a spores-alone control colon and the right image a Fe_3-δ_O_4_-nanoparticle-treated colon. *C*. *difficile* infection-induced inflammation was significantly lower in the Fe_3-δ_O_4_ nanoparticle-treated colon (right panel). (**D**) A histopathological examination of the isolated colon tissue shows prominent infiltration of neutrophils (black arrows in left 400× panel) in the control group, but attenuated inflammation in the Fe_3-δ_O_4_ nanoparticle-treated group (right 400× panel). Neutrophils were morphometrically counted in 10 randomly selected fields in the tissue sections. The number of neutrophils in the control group was 16 ± 5 per 170 m × 130 µm field; the number in the Fe_3-δ_O_4_ nanoparticles-treated group was 2 ± 1 per 170 m × 130 µm field. (**E**) A reverse transcription-quantitative polymerase chain reaction (RT-QPCR) shows that the total RNA extracted from the Fe_3-δ_O_4_-nanoparticle-treated spores expressed significantly lower levels of proinflammatory genes than did the spores in the control group. (*P < 0.05, **P < 0.01; Student’s *t* test) (n = 6).

Before the mouse experiments, the cytotoxicity of Fe_3-δ_O_4_ nanoparticles was evaluated using a methylthiazol tetrazolium (MTT) assay. The colorectal cells were treated with various concentrations of Fe_3-δ_O_4_ nanoparticles (5 to 500 μg/mL) for 24 hours. The viability of C2BBe1 cell line were decreased at the 500 μg/mL of Fe_3-δ_O_4_ nanoparticles. However, the cell viability assay showed no significant cytotoxicity of the Fe_3-δ_O_4_ nanoparticles in HT-29 cell line compared with the control group (Supplementary Fig. 3). To investigate the feasibility of Fe_3-δ_O_4_ nanoparticles as an *in vivo* sporicidal agent, we used a *C*. *difficile* infection mouse model. The inflammatory signal peaked 3 days after *C*. *difficile* infection had been induced (Supplementary Fig. 4). Therefore, in the mouse experiment, we examined the *C*. *difficile* infection pathology 3 days after the mice had been treated with spores. Mice treated with Fe_3-δ_O_4_ nanoparticles plus spores lost significantly less body weight than did the mice treated with spores alone (P = 0.0119, Student’s *t* test) (Fig. 6A). The cecum weight of the Fe_3-δ_O_4_-treated mice was significantly higher than that of the mice treated with spores alone (P = 0.0024, Student’s *t* test) (Fig. 6B), which showed minor inflammation under an *in vivo* imager (P = 0.0406, Student’s *t* test) (Fig. 6C). The histopathological images also showed that neutrophil infiltration was lower in the mice treated with Fe_3-δ_O_4_ nanoparticles plus spores (Fig. 6D) than in the control mice treated with spores alone. A real-time reverse transcription quantitative polymerase chain reaction (RT-qPCR) was used to determine the expression of the proinflammatory genes TNF-α, IFN-γ, and IL-1β, compared with the spores-alone groups (Fig. 6E). The level of inflammation caused by the spores was significantly lower (P = 0.0088 for TNF-α, P = 0.0276 for IFN-γ, and P = 0.0097 for IL-1β, Student’s *t* test) at the molecular level in the mice treated with Fe_3-δ_O_4_ nanoparticles plus spores than in the mice treated with spores alone. To verify whether treatment with 500 μg/mL Fe_3-δ_O_4_ nanoparticles alone induces any adverse side effects *in vivo*, we fed the mice with nanoparticles alone and monitored the biochemistry markers (Supplementary Fig. 5). Based on the ratio of *Firmicutes* to *Bacteroidetes*, we found this nanoparticle does not altered the gut microbiota (Supplementary Fig. 6A), suggesting that this particle did not has the toxicity to the mice.

## Discussion

Based on our review of the literature, this is the first study which shows Fe-based nanoparticles that strongly inhibited the germination of *C*. *difficile* spores despite the growth inhibitory effect of other type of iron oxide nanoparticles to other bacteria vegetative cells was recently reported.

Iron oxide nanoparticles have existed in the earth’s stratum and some organisms^35^. Due to the iron oxide nanoparticles are widely used in medical applications. The iron oxide nanoparticles not only can be the MRI T2 contrast material but also be conjugated to other dyes as detection reagents for different imaging systems^35^. The iron oxide nanoparticles could be used as hyperthermia-based cancer therapeutic reagent when combined to radiofrequency. The iron oxide nanoparticles could even be modified with different materials to become the multi-functional nanoparticles, e.g., targeting, drug releasing, and imaging abilities^36^. We performed the new function of iron oxide nanoparticles that could be a *C*. *difficile* spore germination inhibition reagent in the study. The 22-nm-Fe_3-δ_O_4_ nanoparticles presented as unique octahedron single crystals, and the ratio of iron to oxide was 2.966:4, similar to our previously reported 6-nm Fe_3_O_4_ nanoparticles^30^. The differences between Fe_3-δ_O_4_ and Fe_3_O_4_ include size, surface chemistry, and the presence of minor non-oxidized Fe voids in the Fe_3-δ_O_4_ particles. There was no significant difference between the inhibitory ability of the 22-nm and the 14-nm Fe_3-δ_O_4_ doses. The amount of DPA released from the spores was significantly lower in the Fe_3-δ_O_4_-treated group than in the positive controls. The transition of spores from bright to dark can be observed with a phase-contrast microscope after the DPA has been released^37^. In the present study, because Fe_3-δ_O_4_ nanoparticles inhibited spore germination, there was no transition of spores into phase-dark. However, DPA could be detected, but not in as great abundance as with the taurocholate-treated spores, after the spores had been exposed to Fe_3-δ_O_4_ nanoparticles. Thus, it is conceivable that there is an underlying mechanism that enables Fe_3-δ_O_4_ nanoparticles both to induce DPA release and to inhibit spore germination. We hypothesized that taurocholate can be caught by Fe_3-δ_O_4_ nanoparticles and block the interaction between taurocholate and CspC. CspC is a bile salt germination receptor in *C*. *difficile* and transmits the signal to the downstream signaling molecule. However, we later discovered that taurocholate does not bind with Fe_3-δ_O_4_ nanoparticles, and that taurocholate supernatant without Fe_3-δ_O_4_ nanoparticles was able to trigger *C*. *difficile* spore germination. Therefore, we now hypothesize that Fe_3-δ_O_4_ nanoparticles inhibit bacterial growth without directly interacting with taurocholate. We also hypothesize that Fe_3-δ_O_4_ nanoparticles do not penetrate spores based on our cryo-TEM Supplementary Video 1. The details of the molecular mechanisms of Fe_3-δ_O_4_ nanoparticles require additional study.

Zero-valent iron (ZVI) has recently been reported^38^ to be involved in the selective inhibition of cancerous cells while sparing noncancerous cells because it depletes mitochondrial membrane potential. Because mitochondria share distinct structural and biochemical similarities as well as evolutionary links to bacteria, we hypothesize that the nonstoichiometric non-oxidized Fe of Fe_3-δ_O_4_ also inhibits spore germination. In fact, one study^25^ reported a prominent bactericidal effect of ZVI-based nanoparticles on *E*. *coli* because they effectively caused oxidative damage to bacterial membranes. Our study showed that Fe_3-δ_O_4_-treatment significantly inhibited the germination of *C*. *difficile* spores by damaging spore membranes but not the vegetative cells and therefore did not damage the balance of intestinal normal flora. Thus the specific activity to spore germination must go through other yet to be discovered mechanisms.

It is well known that the expression of the *tcdA* and *tcdB* genes is closely associated with the virulence of *C*. *difficile* infection. We found that Fe_3-δ_O_4_ nanoparticles inhibited germination in both the tcdA(−)tcdB(−) strain and the more virulent tcdA(+)tcdB(+) strain. In an *in vivo C*. *difficile* infection mouse experiment, we found that Fe_3-δ_O_4_ significantly reduced the inflammation indices in the groups treated with spores +Fe_3-δ_O_4_, and that it effectively inhibited *C*. *difficile* spore germination, thus controlling *in vivo* the virulence of *C*. *difficile*. Intriguingly, treatment with 500 μg/mL of Fe_3-δ_O_4_ nanoparticles did not kill vegetative cells.

Because antibiotics are overused everywhere in the world, multidrug-resistant microbes have emerged as a major public health threat^39^. Nanomedicines have inspired various new strategies to control microbes without allowing them to develop drug resistance^40^. Bacterial spores are even more resistant than are the bacteria themselves to harsh environments and antibiotics, but nanoparticle-spore interaction and potential clinical applications are rarely discussed. The germination of *Bacillus subtilis* spores is greatly compromised by treatment with polyionic polymers and then silica nanoparticles^41^. Such treatment, however, does not affect spore viability. We also found that excess PSMA alone did not significantly inhibit the germination of spores; therefore, PSMA must not be important for inhibiting spore germination. In the present study, 500 μg/mL of Fe_3-δ_O_4_ nanoparticles were sufficient to significantly, and almost as efficaciously as 3% sodium hypochlorite, inhibit spore germination, which indicated the potential of Fe_3-δ_O_4_ nanoparticles for clinical development as a novel and effective treatment for managing and perhaps even preventing *C*. *difficile* infection.

Many studies showed their materials existed excellent antibacterial activity, but sometimes the impact of the materials on the normal flora and biocompatibility were rarely discussed. The guts normal flora plays a vital role in animals, because it attend the metabolism and pathogenic colonization prevention in the host^29^. Therefore, we should have to understand the potential adverse effect of Fe_3-δ_O_4_ nanoparticles on normal flora and biocompatibility. We found that Fe_3-δ_O_4_ nanoparticles at a concentration of 500 μg/mL inhibited spore germination but did not damage their vegetative cells. Another study^42^ reported that a high concentration (3 mg/mL) of Fe_3_O_4_ nanoparticles inhibited the growth of *Staphylococcus aureus*. After mice were fed with Fe_3-δ_O_4_ nanoparticles for 72 hours, RT-qPCR was used to analyze the results of normal flora population changes. The two main phyla of bacteria in the gut are *Firmicutes* and *Bacteroidetes* ^43^. We found that the Fe_3-δ_O_4_ nanoparticles did not change the ratio of *Firmicutes* to *Bacteroidetes* in the mice (Supplementary Fig. 6A). *Bacteroides fragilis* and *Enterococcus faecalis* are the common bacteria in the large intestine^44, 45^. Our results showed that Fe_3-δ_O_4_ nanoparticles did not cause damage to the two bacteria (Supplementary Fig. 6B). According to our results we hypothesize that Fe_3-δ_O_4_ nanoparticles do not cause imbalance of gut microbiota. The Fe_3-δ_O_4_ nanoparticles will not be an issue in the normal flora destruction after administering to human and other animals. We also found excellent biocompatibility between Fe_3-δ_O_4_ nanoparticles and a variety of colorectal cells. We previously reported^30, 31^ that Fe_3-δ_O_4_ nanoparticles used with MRI scans and cancer therapy were safe. Although the safe dosages are different in these three studies, we have shown that Fe_3-δ_O_4_ nanoparticles are biocompatible and safe *in vivo*.

Metronidazole and vancomycin are antibiotics frequently used to treat patients with *C*. *difficile* infection, but the some clinical isolates are metronidazole-resistant^46^. Antibiotics unselectively damage pathogens as well as normal flora and might trigger a highly contagious state of super-shedding *C*. *difficile* spores, thus increasing the risk of infection^47^. Spores can survive for many years in harsh environments and wait to be ingested by animals^48^. The imbalance of normal flora and spore germination might be the major cause of a *C*. *difficile* infection relapse. Moreover, bowel inflammation has long been recognized as an important index for *C*. *difficile* infection severity and prognosis. Therefore, we quantitatively assessed *C*. *difficile* infection by directly injecting mice with spores, which is most relevant to the pathogenesis of clinical *C*. *difficile* infection. Transgenic mice with NF-κB-dependent luciferase reporter enabled us to visualize bowel inflammation using the IVIS imaging system. Because of their unique ability to selectively kill spores without damaging normal flora, mammalian cells, or the mouse gastrointestinal tract, Fe_3-δ_O_4_ nanoparticles offer a new strategy for controlling infectious clinical diseases. Fe_3-δ_O_4_ nanoparticle treatment not only inhibited the germination of different clinical strains of *C*. *difficile* spores *in vitro*, but also reduced the virulence of spores that induced *C*. *difficile* infection *in vivo*. We also report the first relatively low-dose but efficacious Fe-based nanoparticle inhibition of spore germination, one which uses a novel mechanism that does not interfere with the growth of healthy vegetative flora, which is an important clinical consideration in *C*. *difficile* infection management. We hypothesize that coating the surfaces of medical devices with Fe_3-δ_O_4_ nanoparticles will reduce the threat of spores in hospitals. Additional research on improving the design of Fe_3-δ_O_4_ nanoparticles should help nanoparticle-based therapeutics become safer and more effective, and should contribute to the development of new therapeutics for *C*. *difficile* infection and other emerging infectious diseases.

## Methods

### Ethics statement and animals

All NF-κB-dependent reporter mice (FVB/NJNarl genetic background) were obtained from the National Laboratory Animal Center in Taiwan and housed in a pathogen-free barrier facility. All animal experiments were approved and in accordance with the relevant guidelines and regulations required by the Institutional Animal Care and Use Committee of National Cheng Kung University (NCKU-IACUC-102-296).

### Bacterial culture and spore purification

*C*. *difficile* CCUG 37780 (tcdA^−^, tcdB^−^) and CCUG 19126 (tcdA^+^, tcdB^+^) were purchased from the Culture Collections of the University of Göteborg (Göteborg, Sweden), and BAA-1805 (tcdA^+^, tcdB^+^) from American Type Culture Collection (Manassas, VA). All strains were incubated in brain-heart infusion-supplemented medium (BHIS; BD Difco, Franklin Lakes, NJ), with 0.5% yeast extract (BD Difco) and 0.1% L-cysteine (Amresco, Solon, OH), at 37 °C under anaerobic conditions. The spores were prepared and purified as previously described^32^ with a slight modification. Briefly, *C*. *difficile* in BHIS medium was diluted in fresh BHIS medium to an optical density (OD) (600 nm) of 0.2. The diluted bacterial suspension (900 μL) was added to a 6-well dish with BHIS agar, and then the dish was incubated at 37 °C in an anaerobic jar (Thermo Fisher, Oxoid Ltd., Basingstoke, England) for 4 days. The whole cells were washed 5 times with ice-cold sterile water, the bacteria were resuspended with 3 mL of ice-cold sterile MQ water. The suspension was spread on top of a 10-mL 50% (wt/vol) sucrose solution (J.T. Baker Chemical, Phillipsburg, PA) in a centrifuge tube, and then centrifuged at 3500 *g* for 20 minutes to separate spores from vegetative cells. The purified spores at the bottom of the centrifuge tube were washed 5 times with ice-cold sterile water to remove sucrose, and then stored at 4 °C.

### Preparation of the nanoparticles

Fe_3-δ_O_4_ nanoparticles (22 nm and 14 nm) were synthesized using thermal decomposition, as previously described^30^. Briefly, 1.42 g of iron acetylacetonate was mixed with 0.57 mL of oleic acid and 20 mL of trioctylamine (all 3: Sigma-Aldrich, St. Louis, MO). The solution was refluxed at 325 °C in an argon environment for 30 minutes. After the solution had cooled down to room temperature, the precipitates were collected with a magnet and washed 3 times with toluene. The Fe_3-δ_O_4_ nanoparticles were collected with a magnet and then transferred to chloroform solutions (Merck, Whitehouse Station, NJ) containing 0.4 mg/mL of PSMA (Sigma-Aldrich) and were left there for 6 hours at 55 °C. The Fe_3-δ_O_4_ nanoparticles were collected, washed 3 times with MQ water, and then stored at 4 °C.

### Optical density based spore germination test

Spores were germinated as previously described^32^. Before the germination experiments, the spore suspension was incubated at 60 °C for 30 minutes. The heat-treated spores were then moved to ice. The *C*. *difficile* spores (concentration: OD_600_ 0.5) were co-incubated with various nanoparticles or at different concentrations (5 to 500 μg/mL) in BHIS in a 96-well plate for 20 minutes. The concentration of Fe_3-δ_O_4_ nanoparticles was measured based on the total particle weight per 1 mL of solution. Fe_3-δ_O_4_ (22-nm) nanoparticles were used here. Spores treated with 3% bleach (Wako, ﻿Osaka, Japan) were the positive controls to inhibit germination, and spores treated with BHIS only were the negative controls. After the spore + nanoparticle co-incubation, the spores were treated with 10 mM taurocholate (Sigma-Aldrich) to induce germination. The OD_600_ of treated spores was kinetically determined at 1 minute intervals using a spectrophotometer (TECAN, Grödig, Austria) at room temperature for 12 minutes. The OD at different time points was used to plot the spore germination curve.

### Phase-contrast microscopy observation of spore germination

The images of spore germination were recorded using phase-contrast optical microscopy described elsewhere^49^. The purified spores were incubated at 60 °C for 30 minutes. The spores were co-incubated for 20 minutes with 500 μg/mL of Fe_3-δ_O_4_ nanoparticles or with buffer alone. Taurocholate was then added to the samples to a final concentration of 10 mM in BHIS. Five microliters of samples were then dropped onto the 0.7% agarose surface on the glass slide. After 15 minutes, phase-contrast images of the spores were captured (Nikon Eclipse 80i; Tokyo, Japan). Six random fields of view were obtained for each experimental group.

### Dipicolinic acid (DPA) release assay

The DPA release was estimated by modified method from a previous study^50^. Briefly, the spores were heated as described and washed with distilled water then resuspended in spore germination buffer (10 mM Tris [pH 7.5], 150 mM NaCl, and 100 mM glycine). The spores were treated with MQ water or 500 μg/mL of Fe_3-δ_O_4_ nanoparticles for 20 minutes in a black 96-well plate before taurocholate (10 mM) and terbium (III) chloride (TbCl_3_) (100 μM) were added. The total DPA of the spores was extracted by boiling the samples for 30 minutes. The spores treated with TbCl_3_ only were negative controls. The DPA-Tb signal was monitored immediately in real-time using a microplate reader (FlexStation 3 Multi-Mode; Molecular Devices, CA) with excitation/emission at 270 nm and 545 nm, and a cutoff at 530 nm.

### Spore viability analysis

After the spore germination curves had been recorded, each aliquot was removed from the 96-well plate and plated on BHIS agar dishes and serially diluted. The plate-counts (CFU/mL) at each dilution were done after 48 hours of incubation in an anaerobic environment. The inhibition rates were calculated using the following formula:

$$1:[1-({\rm{treatment}}\,\text{CFU}/\text{control}\,{\rm{CFU}})]\times 100 \% .$$

### Nanoparticle-spore binding analysis

*C*. *difficile* BAA-1805 spores at a concentration of OD_600_ 0.5 were co-incubated with Fe_3-δ_O_4_ nanoparticles (500 μg/mL) in BHIS in a 96-well plate for 20 minutes. The spore samples without Fe_3-δ_O_4_ treatment were controls. The samples were placed next to a magnet for 5 minutes and then all supernatant was removed to other tubes. The magnet-attracted parts and supernatant were washed 3 times with 1× phosphate-buffered saline. All samples were resolved in distilled deionized water, and the *tcdB* DNA in samples was detected using a polymerase chain reaction (PCR). Spore and nanoparticle images were captured using a transmission electron microscope (TEM) (JEM-1400; JEOL, Tokyo, Japan).

### *In vivo* analysis of *C. difficile* infection

To directly monitor the colonic inflammation, we infected mice with *C*. *difficile* spores in a previously generated NF-κB-dependent reporter mouse model containing the luciferase transgene under the transcriptional control of NF-κB (NF-κB-RE-luciferase)^51^. Before the mice were fed the spores with NF-κB reporter, they were given an antibiotic cocktail (0.4 mg/mL of kanamycin, 0.035 mg/mL of gentamicin, and 0.057 mg/mL of colistin) in their drinking water for 48 hours and a refreshed cocktail every 24 hours for 2 days. The mice were gavaged with a 200-μL proton pump inhibitor (PPI) (2 mg/mL) every 12 hours for 2 days before the *C*. *difficile* spore infection. *C*. *difficile* CCUG 19126 spores (2 × 10^5^ CFUs) were co-incubated with or without 500 μg/mL of Fe_3-δ_O_4_ nanoparticles for 20 minutes before they were gavaged with 100 L of sample solution. While the spores and Fe_3-δ_O_4_ nanoparticles were incubating, all the mice were gavaged with a 50- μL PPI (2 mg/mL) and then intraperitoneally injected with clindamycin (4 mg/kg). The antibiotic cocktail water was replaced with normal water after the *C*. *difficile* infection. All mice were monitored for *C*. *difficile* infection symptoms, e.g., diarrhea, weight loss, hunched posture, and death. Seventy-two hours post-infection, the mice were intraperitoneally injected with 150 mg/kg of luciferin (PerkinElmer, Waltham, MA) to show NF-κB activation-mediated luminescence. The mice were anesthetized with isoflurane and oxygen, and then images were collected for 5 minutes using an imaging system (Xenogen IVIS^®^ Spectrum; Advanced Molecular Vision, Grantham, Lincolnshire, UK). Data were analyzed (Xenogen Living Image^®^), and luciferase activity was presented in photons/sec/cm^2^/steradian (p/s/cm^2^/sr). After the IVIS images had been obtained, a reagent (TRI; Sigma-Aldrich) was used to extract RNA from colon tissue samples. The levels of inflammatory gene expression were estimated using a real-time PCR assay (StepOnePlus; Applied Biosystems).

### Histopathology examination

Histopathological analysis was used to evaluate *C*. *difficile* infection-induced mucosal damage and inflammation. Resected colon tissue samples were fixed in 4% formaldehyde buffered with PBS and then embedded in paraffin. Deparaffinized 6-μm-thick sections were stained with hematoxylin and eosin. The results were captured using optical microscopy. Neutrophils were randomly counted in 10 fields for both the spores-alone group and the Fe_3-δ_O_4_-treated-spores group.

### Statistical analysis

GraphPad Prism 5.01 was used for all statistical analyses. All experiments were done in triplicate. Data are means ± standard error of the mean (SEM) from three independent experiments. One-way analysis of variance (ANOVA) and then Tukey’s Multiple Comparison test were used for spore germination curve analyses, and Student’s *t* test was used in the other CFU inhibition test.

## Electronic supplementary material

Supplementary Video 1

Supplementary Information
