# Supplementary material for: Octahedron Iron Oxide Nanocrystals Prohibited Clostridium difficile Spore Germination and Attenuated Local and Systemic Inflammation
Source: Sci Rep. 2017 Aug 15;7:8124. doi: 10.1038/s41598-017-08387-y (PMC5558001; doi:10.1038/s41598-017-08387-y)
Supplement: Supplementary file 2 — Supplementary Information [file 41598_2017_8387_MOESM2_ESM.pdf]

**Supplementary Information**

# Octahedron Iron Oxide Nanocrystals Prohibited *Clostridium difficile* Spore Germination and Attenuated Local and Systemic Inflammation

Wei-Ting Lee[1], Ya-Na Wu[2], Yi-Hsuan Chen[3], Shang-Rung Wu[2], Tsai-Miao Shih[2], Tsung-Ju Li[1], Li-Xing Yang[1], Chen-Sheng Yeh[4], Pei-Jane Tsai[1,3,5,*], Dar-Bin Shieh[1,2,6,7,*]

[1]Institute of Basic Medical Sciences, National Cheng Kung University, 1 University Road, Tainan 701, Taiwan.

[2]Institute of Oral Medicine, National Cheng Kung University, 1 University Road, Tainan 701, Taiwan.

[3]Department of Medical Laboratory Science and Biotechnology, National Cheng Kung University, 1 University Road, Tainan 701, Taiwan.

[4]Department of Chemistry, National Cheng Kung University, 1 University Road, Tainan 701, Taiwan.

[5]Center of Infectious Disease and Signaling Research, National Cheng Kung University, 1 University Road, Tainan 701, Taiwan.

[6]Department of Stomatology, National Cheng Kung University Hospital, College of Medicine, National Cheng Kung University, 138 Sheng-Li Road, Tainan 704, Taiwan.

[7]Advanced Optoelectronic Technology Center and Center for Micro/Nano Science and Technology, National Cheng Kung University, 1 University Road, Tainan 701, Taiwan.

[*]Corresponding authors:

Dar-Bin Shieh, Institute of Oral Medicine, National Cheng Kung University, Tainan 70101, Taiwan.

Tel: +886-6-235-3535 ext. 5410; Email: dbshieh@mail.ncku.edu.tw

Pei-Jane Tsai, Department of Medical Laboratory Science and Biotechnology, National Cheng Kung University, 1 University Road, Tainan 701, Taiwan.
Tel: +886-6-235-3535 ext. 5763; Email: peijtsai@mail.ncku.edu.tw

## Methods

*Germination kinetic assay*

Germination kinetic study was estimated based on Sorg and Sonenshein[1]. The purified and heated spores were incubated for 20 minutes in BHIS medium with or without $Fe_{3-\delta}O_4$ nanoparticles (50 µg/mL) in a 96-well dish, and then they were treated with 2-, 5-, 10-, 20-, 40-, or 50-mM taurocholate. Once the taurocholate was added to the aliquots, the germination results were monitored by measuring $OD_{600}$.

*Cell viability assay*

Human C2BBe1 and HT-29 cells ($1 \times 10^4$ each) were cultured overnight with 2% FBS medium (DMEM with 0.01 mg/mL human transferrin [T-0662; Sigma-Aldrich] and 1 mM sodium pyruvate for C2BBe1 cells; RPMI 1640 medium for HT-29 cells) in a humidified atmosphere of 5% $CO_2$ at 37 °C. The cells were incubated for 24 hours with different concentrations of $Fe_{3-\delta}O_4$ nanoparticles, and then cell viability was estimated using an MTT assay and recorded on a spectrophotometer at a wavelength of 550 nm.

*The relationship between PSMA and spore germination*

CCUG 37780 spores were treated for 20 minutes with 10 mg/mL of PSMA, larger than the dose of $Fe_{3-\delta}O_4$. The spores were then treated with 10 mM taurocholate, and spore germination was recorded using a spectrophotometer for 12 minutes at room temperature.

*The interaction between taurocholate and $Fe_{3-\delta}O_4$ nanoparticles*

$Fe_{3-\delta}O_4$ nanoparticles were mixed well for 20 minutes with 40-mM taurocholate, and then the nanoparticles were removed using a magnet. The taurocholate was then added to the CCUG

37780 spores after the $OD_{600}$ of the treated spores had been kinetically recorded using the spectrophotometer.

*The effect* of *$Fe_{3-\delta}O_4$ nanoparticles on mouse colons*

After the mice had been gavaged with 500 µg/mL of $Fe_{3-\delta}O_4$ nanoparticles or autoclaved water for 12 hours or 24 hours, they were intraperitoneally injected with 150 mg/kg of luciferin. They were next anesthetized with isoflurane and oxygen, after which, colonic images were obtained using the IVIS® Spectrum *in vivo* imaging system. The body weights of the mice and the weights of the cecums were recorded.

*The effect* of *$Fe_{3-\delta}O_4$ nanoparticles on guts microbiota*

The *Firmicutes*/*Bacteroidetes* ratio was estimated by the real-time PCR analysis following the previous study[2]. After the mice had been gavaged with 500 µg/mL of $Fe_{3-\delta}O_4$ nanoparticles or water for 72 hours, the stools were collected freshly and stored at 4°C under anaerobic conditions. The DNA in the stools was extracted via the High Pure PCR Template Preparation Kit (Roche). The all real-time PCR reactions contained 4 ng extracted DNA, primers and SYBR Green master mix (Thermo Fisher Scientific).

*The viability of gut common bacteria test*

*Bacteroides fragilis* (ATCC 25285) and *Enterococcus faecalis* (ATCC 29212) were purchased from American Type Culture Collection (Manassas, VA). *B. fragilis* were incubated on CDC blood agar plates (BD Difco) and *E. faecalis* were incubated on TSB blood agar plates (BD Difco). Both of the two bacteria were incubated at 37 °C under anaerobic conditions. After activation from the frozen tube, the bacteria were co-incubated with 500 µg/mL of $Fe_{3-\delta}O_4$ nanoparticles or water for 20 minutes and then plated on the blood agar plates. The plate-counts at each dilution were recorded after 48 hours of incubation under the anaerobic environment. The viability rates were calculated with the following formula:

1: (treatment CFU/control CFU) $\times$ 100%

*Cryo-electron tomography video*

Purified BAA-1805 spores were treated for 20 minutes with 50 μg/mL of $Fe_{3-\delta}O_4$ nanoparticles before the spores were dropped onto a glow-discharge copper grid with holey carbon film (01800-F; Ted Pella, Redding, CA). The grid with spores was frozen in liquid ethane using a semi-automated plunge-freezing instrument (Cryoplunge 3; Gatan, Pleasanton, CA). Cryo-electron tomography was done using the TEM (JEOL) with 200 kV field emission guns and a 1024 × 1024 CMOS camera. The single tilt series of cryo-specimens was obtained from −60.0 to +60.0 degrees (3-degree angular increment and 0.83-nm pixel size). Whole images were aligned in IMOD 4.7 (http://bio3d.colorado.edu/imod/).

**References**

1. Sorg, J. A. & Sonenshein, A. L. Inhibiting the initiation of Clostridium difficile spore germination using analogs of chenodeoxycholic acid, a bile acid. *J Bacteriol* **192**, 4983-4990 (2010).
2. Pedersen, R., Andersen, A. D., Mølbak, L., Stagsted, J. & Boye, M. Changes in the gut microbiota of cloned and non-cloned control pigs during development of obesity: gut microbiota during development of obesity in cloned pigs. *BMC Biol.* **13**, 1 (2013).

Supplementary Table 1 $Fe_{3-\delta}O_4$ affected the *C. difficile* CCUG 37780 spore kinetic germination.

| Treatment | $K_m$ (µM) | $V_{max}$ | $K_i$ (µg/mL) |
|---|---|---|---|
| Control | 4.34 | 0.014 | |
| $Fe_{3-\delta}O_4$ | 8.43 | 0.017 | 62.44 ± 28.25 |

$K_m$ = Michaelis constant; $Ki$ = inhibiting constant.

Formula 2: $K_i$ = [50 µg/ml $Fe_{3-\delta}O_4$ NPs]/($K_m$ taurocholate – 1)

The control set stands for the CCUG 37780 spores with taurocholate only. The spores were treated with different doses of taurocholate and 50 µg/mL of $Fe_{3-\delta}O_4$ nanoparticles. The results were recorded spectrophotometrically at 600 nm.

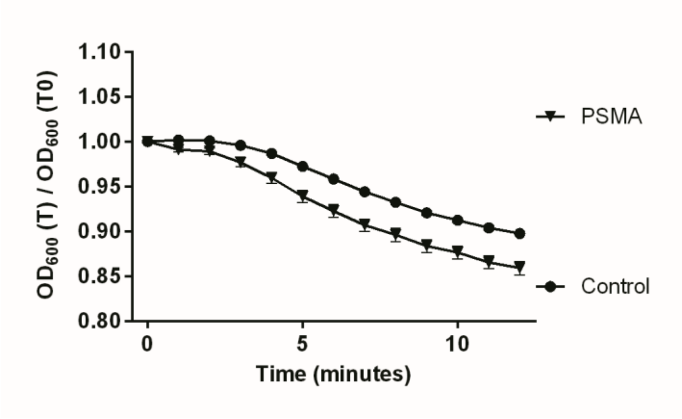


**Supplementary Figure 1. Germination was not inhibited in *C. difficile* spores incubated with the polymer PSMA.** After 10 mg/mL of PSMA (▼) had been incubated for 20 minutes with CCUG 37780 spores, taurocholate was added to induce spore germination. The PSMA-treated spores still germinated after the taurocholate treatment.

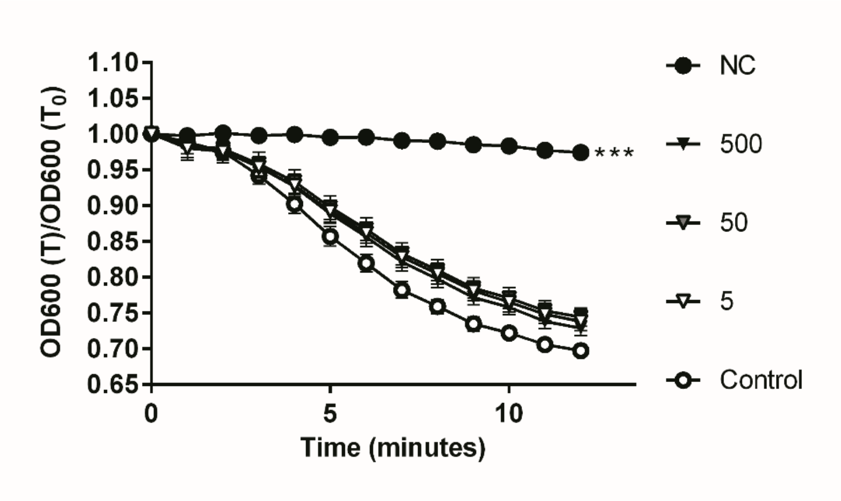


**Supplementary Figure 2. $Fe_{3-\delta}O_4$ nanoparticles did not interact with taurocholate.** $Fe_{3-\delta}O_4$ nanoparticles and taurocholate were thoroughly mixed for 20 minutes The $Fe_{3-\delta}O_4$ nanoparticles were separated using a magnet. The CCUG 37780 spores were treated with the separated taurocholate to initiate germination. The nanoparticle-pretreated taurocholate solution induced spore germination; thus, the nanoparticles did not interact with the taurocholate. NC: spores were not treated with taurocholate. (***$P < 0.001$, one-way analysis of variance (ANOVA) and then Tukey's Multiple Comparison test)

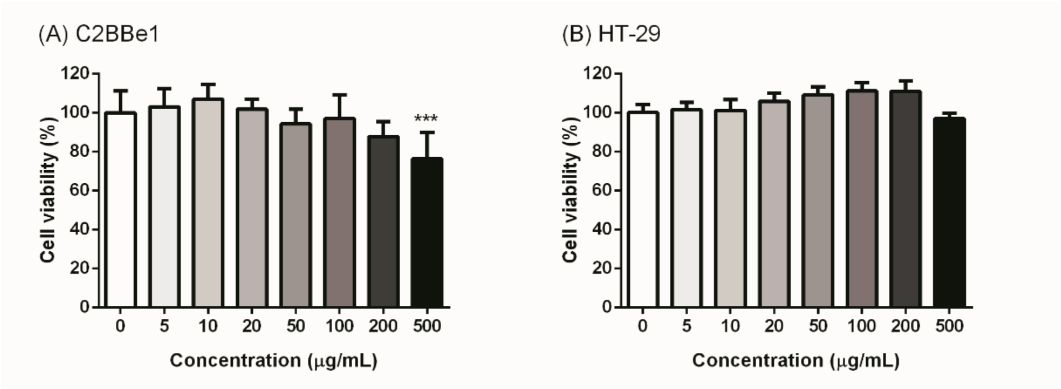


**Supplementary Figure 3. Cell viability was not affected by treatment with $Fe_{3-\delta}O_4$ nanoparticles.** (A) C2BBe1 and (B) HT-29 colorectal cells were cultured for 24 hours (C2BBe1: DMEM; HT-29: RPMI 1640 medium) with 2% FBS and treated with various doses of $Fe_{3-\delta}O_4$ nanoparticles for 24 hours. An MTT assay showed that $Fe_{3-\delta}O_4$ nanoparticle treatment had affected cell viability of CeBBe1 at the concentration of 500 μg/mL but not affected HT-29 cells. (***$P < 0.001$, one-way analysis of variance (ANOVA) and then Dunnett's multiple comparisons test)

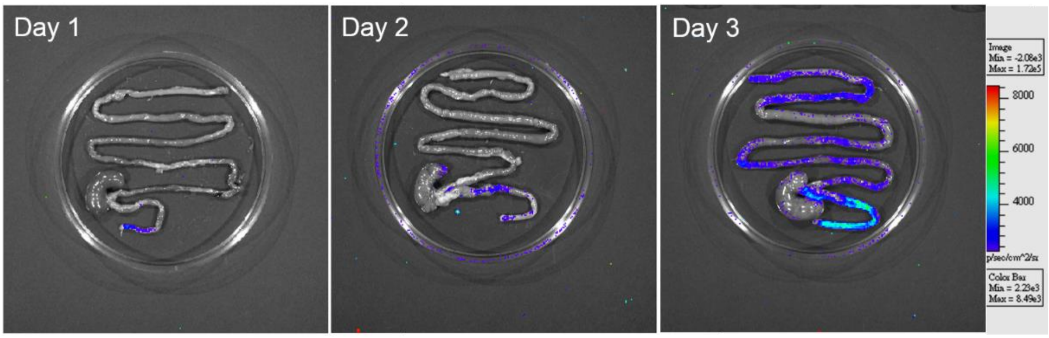


**Supplementary Figure 4. Estimating purified CCUG 19126 spore-induced colitis in mice.** The colon was collected and spore-induced colitis was estimated using IVIS. The inflammation signal level was substantially higher on day 3 in *C. difficile* infected mice. Therefore, we analyzed the results of mouse experiments on the 3rd day after *C. difficile* infection.

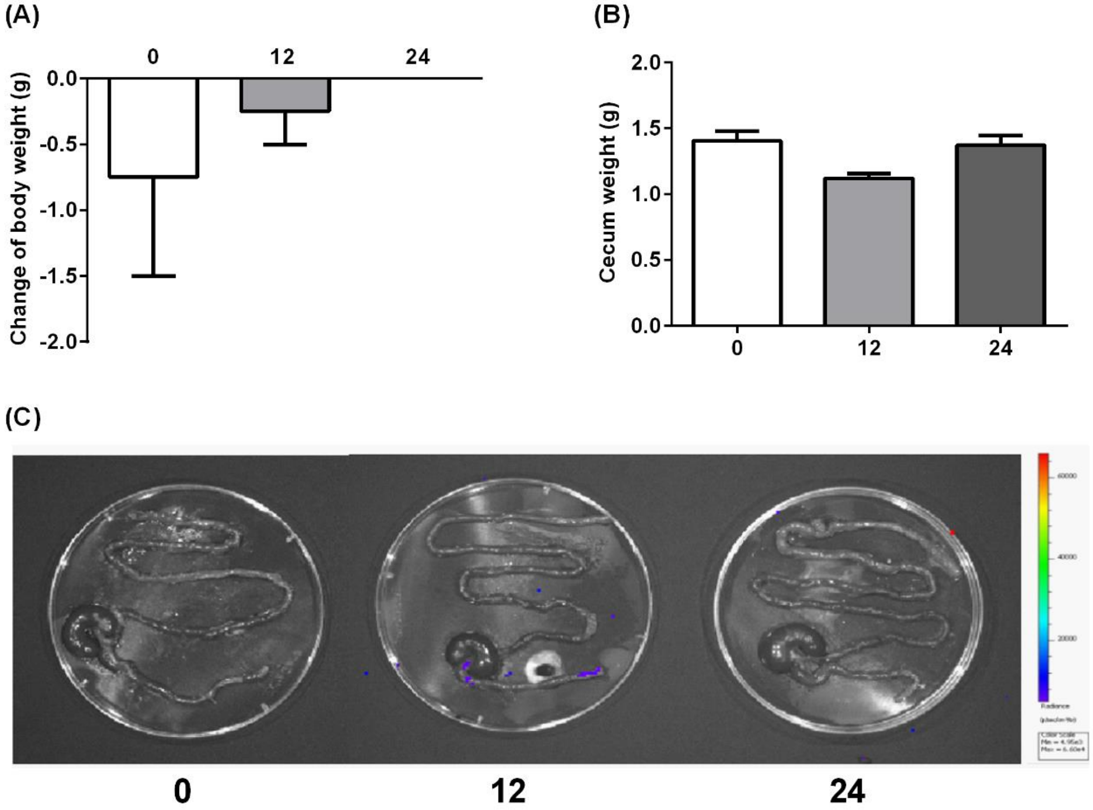


**Supplementary Figure 5. Mice were not adversely affected by treatment with $Fe_{3-\delta}O_4$ nanoparticles.** (A) The mean bodyweight-loss of the three groups was not significantly different. (B) 500 µg/mL $Fe_{3-\delta}O_4$ treatment at different time points did not affect the isolated mouse cecums. (C) Twelve hours after the mice had been treated with $Fe_{3-\delta}O_4$ nanoparticles, their colons were collected. Twelve hours later, their colitis was estimated using IVIS. The mice treated with $Fe_{3-\delta}O_4$ nanoparticles produced no inflammation signal which indicated that colitis had been induced.

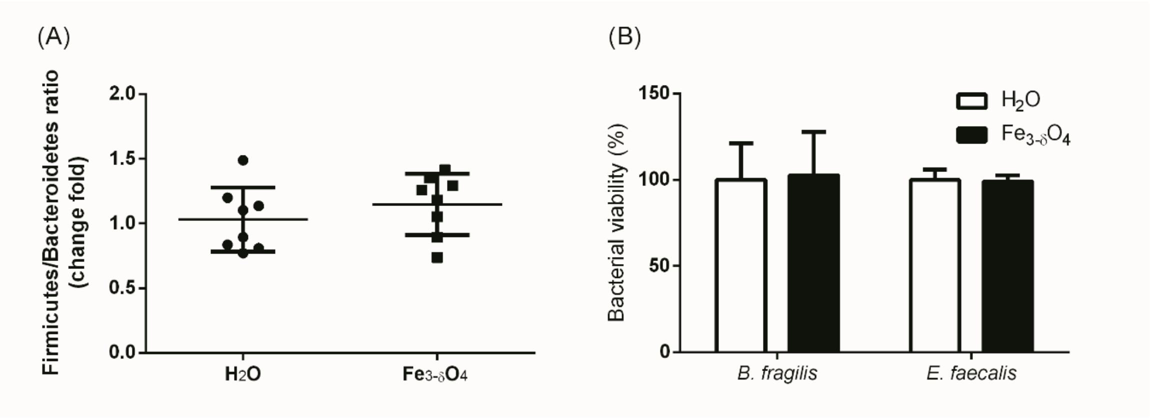


**Supplementary Figure 6. The gut microbiota and gut common bacteria were not affected by $Fe_{3-\delta}O_4$ nanoparticles (500 μg/mL).** (A)The *Firmicutes* to *Bacteroidetes* ratio in the guts of $Fe_{3-\delta}O_4$ nanoparticles-treated mice was similar to the control mice. (B) Both of *B. fragilis* and *E. faecails* were not damaged by the $Fe_{3-\delta}O_4$ nanoparticles-treated. The results indicated that the $Fe_{3-\delta}O_4$ nanoparticles could not decrease gut common bacteria viability.

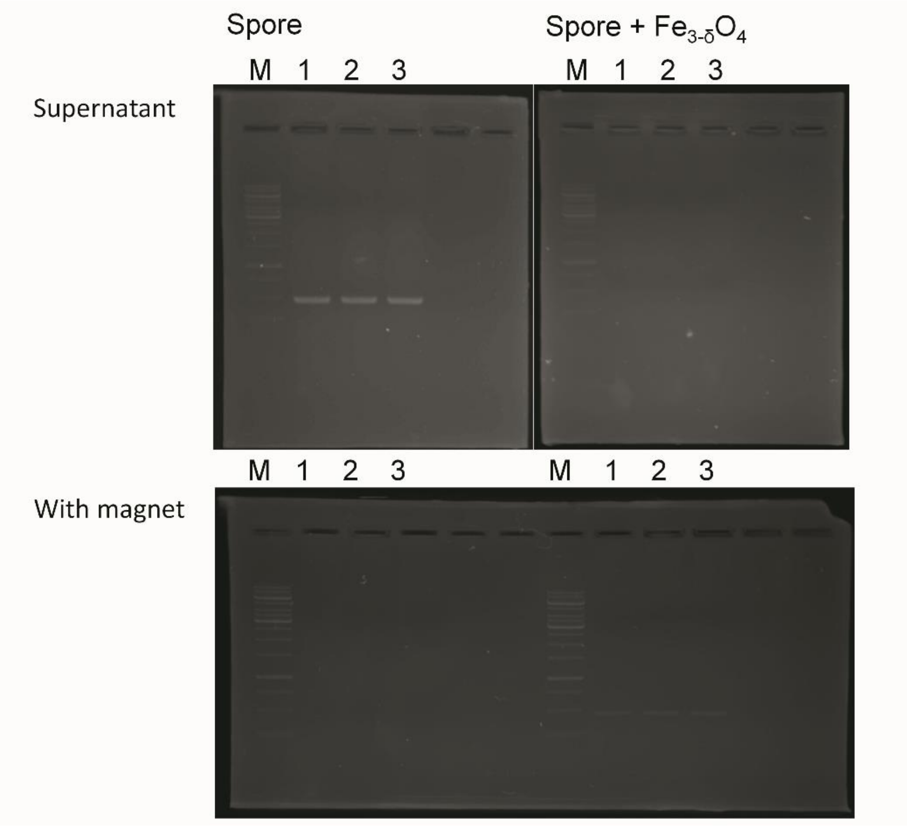


**Supplementary Figure 7. The full-length gels of figure 5B.** After the spores were incubated with/without $Fe_{3-\delta}O_4$ nanoparticles (500 µg/mL) for 20 minutes, the *tcdB* genes from the supernatant or the magnetically concentrated precipitates were detected after PCR amplification. Lanes 1-3 are spores alone and lanes 4-6 are spores treated with $Fe_{3-\delta}O_4$ nanoparticles. M is the GeneRuler 1kb DNA ladder marker (Thermo Fisher).

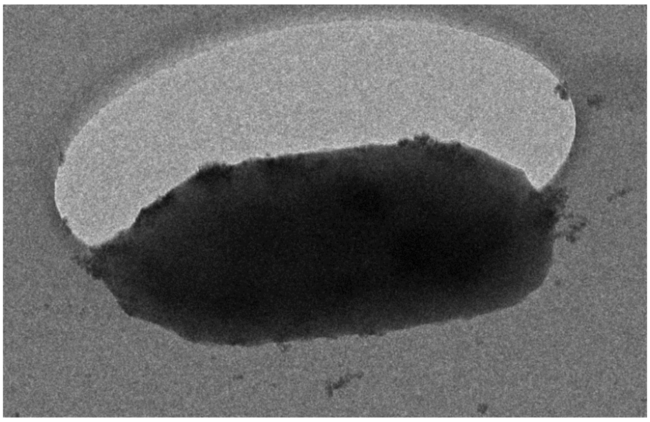

**Supplementary Video 1. CryoEM showed that the $Fe_{3-\delta}O_4$ nanoparticles had bound with the purified BAA-1805 spores.** TEM images (Fig. 5C) do not show whether the $Fe_{3-\delta}O_4$ nanoparticles have merely bound to the surface of the spores or penetrated the spores. Cryo-EM tomography using a different angular tilt showed that $Fe_{3-\delta}O_4$ nanoparticles had bound only to the surface of the spores but had not penetrated them.
